# Scropolioside B Inhibits IL-1***β*** and Cytokines Expression through NF-***κ***B and Inflammasome NLRP3 Pathways

**DOI:** 10.1155/2014/819053

**Published:** 2014-10-16

**Authors:** Tiantian Zhu, Liuqiang Zhang, Shuang Ling, Ju Duan, Fei Qian, Yiming Li, Jin-Wen Xu

**Affiliations:** ^1^Murad Research Institute for Modernized Chinese Medicine, Shanghai University of Traditional Chinese Medicine, Shanghai 201203, China; ^2^School of Pharmacy, Shanghai University of Traditional Chinese Medicine, Shanghai 201203, China

## Abstract

Chronic inflammation is associated with various chronic illnesses including immunity disorders, cancer, neurodegeneration, and vascular diseases. Iridoids are compounds with anti-inflammatory properties. However their anti-inflammatory mechanism remains unclear. Here, we report that scropolioside B, isolated from a Tibetan medicine (*Scrophularia dentata* Royle ex Benth.), blocked expressions of TNF, IL-1, and IL-32 through NF-*κ*B pathway. Scropolioside B inhibited NF-*κ*B activity in a dose-dependent manner with IC_50_ values of 1.02 *μ*mol/L. However, catalpol, similar to scropolioside B, was not effective in inhibiting NF-*κ*B activity. Interestingly, scropolioside B and catalpol decreased the expression of NLRP3 and cardiolipin synthetase at both the mRNA and protein level. Our results showed that scropolioside B is superior in inhibiting the expression, maturation, and secretion of IL-1*β* compared to catalpol. These observations provide further understanding of the anti-inflammatory effects of iridoids and highlight scropolioside B as a potential drug for the treatment of rheumatoid arthritis and atherosclerosis.

## 1. Introduction

Acute inflammatory responses are essential for pathogen control and tissue repair and can also cause severe tissue damage. During chronic infections and age-associated immune dysregulation, inflammatory processes may induce a variety of harmful effects on an organism [1]. Chronic inflammation is associated with chronic illnesses including cancer, neurodegeneration, and vascular diseases [2–4]. Infection or cell damage triggers the release of proinflammatory cytokines such as interleukin- (IL-) 1*β* and tumor necrosis factor- (TNF-) *α*, which are key mediators of the host immune response. Signal transduction of inflammatory cytokines includes ligands, receptors, coreceptors, and cytosolic and nuclear signaling mechanisms. These mechanisms can activate the NF-*κ*B, JNK, p38 MAPK, STAT, and PI3K signaling pathways [5]. On the other hand, inflammasomes play a key role in the regulation of inflammation and immune responses by producing proinflammatory cytokines [6]. Studies have shown that inflammasomes are involved in atherosclerosis [7], metabolic syndrome [8], type 2 diabetes [9], alcoholic steatohepatitis [10], mucosal immune response [11], rheumatoid arthritis [12], and gout [13]. The nucleotide-binding domain- (NOD-) like receptor protein 3 (NLRP3) inflammasome is a multiprotein complex that regulates the maturation of proinflammatory cytokines IL-1*β* and IL-18. It consists of NOD-like receptor, NLRP3, the adaptor protein ASC (apoptosis-associated speck-like protein containing caspase-1 activator domain, CARD), and caspase-1. Upon exogenous and endogenous stimuli, the NLRP3 inflammasome forms through activation of NLRP3 and recruitment of ASC and pro-caspase-1, resulting in caspase-1 activation and subsequently processing of pro-IL-1*β* and pro-IL-18 into their active forms [14].

Iridoid is derived from Scrophulariaceae, Rubiaceae, Labiatae, Gentianaceae, Oleaceae, and so on; it is mainly derived from* Scrophularia* L. [15]. The iridoids are comprised of a large family of distinctive bicyclic monoterpenes that possess a wide range of pharmacological properties, including anticancer, anti-inflammatory, antifungal, and antibacterial activities [16, 17]. Scropolioside A exhibited anti-inflammatory properties against different experimental models of delayed-type hypersensitivity. Scropolioside A also inhibited the production of prostaglandin E2, leukotriene B4, nitric oxide, and some interleukin but had no effect on the production of IL-10. Moreover, it modified the expression of both nitric oxide synthase-2 and cyclooxygenase-2, as well as the activation of NF-*κ*B in RAW 264.7 macrophages [18]. Scropolioside D also possessed significant antidiabetic and anti-inflammatory activity [19]. However, although scropolioside B exhibited moderate antibacterial activity against strains of multidrug and methicillin-resistant* Staphylococcus aureus* (MRSA) and a panel of rapidly growing mycobacteria with minimum inhibitory concentration (MIC) values ranging from 32 to 128 *μ*g/mL [20], it had no significant effect on TXB2-release [21]. We previously reported different anti-inflammatory effects of other iridoid components [22, 23].* Scrophularia dentata* Royle ex Benth. in Tibet is used for antiviral and anti-inflammatory treatment. Therefore, in this study, we examined scropolioside B isolated from* S. dentata* Royle ex Benth. We determine whether scropolioside B exhibits anti-inflammatory effect and further analyze its underlying mechanism in human monocytes.

## 2. Materials and Methods

### 2.1. Cell Cultures and Reagents

Human Embryonic Kidney 293 cells (HEK293 cells) were purchased from Yongzheng Grubber Products Corporation (Nanjing, China) and THP-1 cells were from the Chinese Academy of Sciences (Shanghai, China). Cells were cultured on 100 mm tissue culture dishes or 100 mL flasks in Dulbecco's modified Eagle's medium (DMEM) containing 10% fetal bovine serum (Gibco, Invitrogen, USA) at 37°C in a humidified incubator under 5% CO_2_ and 95% air. During experiments, the cells were plated in 24-well plates or 30 mm tissue culture dishes for 16 or 24 h.

### 2.2. Extraction and Isolation of Scropolioside B

NMR spectra were acquired using a Bruker AM-400 spectrometer. ESI-MS and HR-ESI-MS were obtained using an Esquire 3000 plus and a Q-TOF-Ultima mass spectrometer, respectively. Silica gel (200 mesh to 300 mesh; Qingdao Haiyang Chemical Co., Ltd., Qingdao, China), C_18_ reversed-phase silica gel (150 to 200 mesh; Fuji Silysia Chemical, Ltd., Aichi, Japan), MCI gel (CHP20P, 75 *μ*m to 150 *μ*m; Mitsubishi Chemical Industries, Ltd., Tokyo, Japan), and Sephadex LH-20 gel (Pharmacia Biotech AB, Uppsala, Sweden) were used for column chromatography (CC).

*Plant Material*. Whole plants of* S. dentata* Royle ex Benth. were collected from Lhasa, Tibet, China, in October 2010. The plant was identified by Professor Zhili Zhao (School of Pharmacy, Shanghai University of Traditional Chinese Medicine). The voucher specimen (number CX2010) was deposited at the Herbarium of the Department of TCM Chemistry, School of Pharmacy of Shanghai University of Traditional Chinese Medicine (Shanghai, China).

### 2.3. Luciferase Assay

To assay NF-*κ*B promoter activity, THP-1 cells were transiently transfected with a luciferase reporter gene. pNF-*κ*B-TA-Luc was purchased from Stratagene (USA). Cells were plated one day prior to transfection so that cells will be approximately 80% confluent on the day of transfection. On the day of transfection, DNA was diluted to 2 *μ*g per 100 *μ*L of serum-free medium, and an appropriate amount of FUGENE HD Transfection Reagent (Promega, USA) was added to achieve the proper ratio of reagent to DNA. The mixture was incubated for 0–15 minutes, and 100 *μ*L was added to each well to be transfected. Cells were transfected for 5 hours before changing to fresh media. One hour after transfection, TNF-*α* was added to the cells for 16–20 hours. Luciferase activity was measured in the cell lysates using the Promega Luciferase Assay System according to the manufacturer's instructions (Promega, USA).

### 2.4. Quantitative Real-Time PCR (qRT-PCR)

Total RNA was extracted using TRIzol (Life Technology, Carlsbad, CA, USA) according to the manufacturer's instructions. Real-time PCR amplification and detection were performed using the SYBR Green qPCR SuperMix-UDG with ROX (Life Technologies) in a fluorescence thermal cycler (StepOne Real-Time PCR system, Life Technologies) according to the manufacturer's protocol. Gene expression was normalized using GAPDH as a reference gene. Relative mRNA expression levels were calculated following the ΔΔCt method with the following primers: GAPDH, IL-1*β*, TNF-*α*, IL-32*β*, IL-32*γ*, CLS1, and NLRP3 in Table 1. All amplifications were conducted within the linear range of the assay and normalized to respective GAPDH levels using SPSS Version 18.0 (SPSS Institute, Inc., Chicago, IL, USA).

### 2.5. Western Blot

After treatment, cells were centrifuged and lysed in Triton/NP-40 lysis buffer containing 0.5% Triton X-100, 0.5% Nonidet P-40, 10 mmol/L Tris pH 7.5, 2.5 mmol/L KCL, 150 mmol/L NaCl, 20 mmol/L *β*-glycerophosphate, 50 mmol/L NaF, and 1 mmol/L Na_3_VO_4_, sonicated by JY92-2D ultrasonic homogenizer (NingBo Scientz Biotechnology Co., Ltd., Zhejiang, China), and then centrifuged for 10 min at 10000 g. The supernatant was analyzed for protein concentration using a protein assay kit (Bio-Rad, Hercules, CA, USA), and equal amounts of protein (30 *μ*g/sample) were separated by SDS-PAGE and blotted onto nitrocellulose membranes (Pall China, Shanghai, China). The blots were blocked overnight with 5% nonfat dried milk in a buffer containing 140 mmol/L NaCl, 20 mmol/L Tris-HCl at pH 7.5, and 0.1% Tween-20 and incubated with the following primary antibodies: NLRP3 Rabbit mAb (Cell Signaling Technology, Beverly, MA, USA), IL-1*β* Mouse Monoclonal IgG_20_ (Santa Cruz Biotechnology, Dallas, TX, USA), and HRP-conjugated Monoclonal Mouse Anti-GAPDH (KangChen Bio-tech, Shanghai, China) incubated at 4°C with gentle shaking, overnight. The secondary antibody was conjugated with horseradish peroxidase. The membrane was exposed to high performance autoradiography film (Fuji Film Corp., Tokyo, Japan) and visualized using the ECL Immobilon Western chemiluminescent HRP substrate (WBKLS0500) (Millipore, USA). Quantitative analysis was performed by Quantity One software. Western blot experiments were performed in triplicate.

### 2.6. ELISA

The culture medium from the control and treated cells was collected, centrifuged, and stored at −80°C until tested. IL-1*β* was measured using Abcam Human ELISA Kit (Abcam, Cambridge, England) according to the manufacturer's instructions. Standard or sample was added to each well and incubated for 2.5 h at room temperature. The prepared biotin antibody was then added to each well, followed by incubation for 1 h at room temperature. Streptavidin solution was added and incubated for 45 minutes at room temperature. Finally, TMB One-Step Development Solution was added to each well and incubated for 30 minutes at room temperature. A stop solution was then added to each well and read at 450 nm immediately.

### 2.7. Date Analysis

Each experiment was performed at least 3 times. The results were presented as means ± standard error of mean (SD). All data was analyzed using SPSS software, and one-way ANOVA was used to determine the statistical significance of differences between the means. Differences were statistically significant when *P* < 0.05.

## 3. Results

### 3.1. Blocking IL-1*β* and TNF-*α* Expression by Scropolioside B

**Figure 1:**
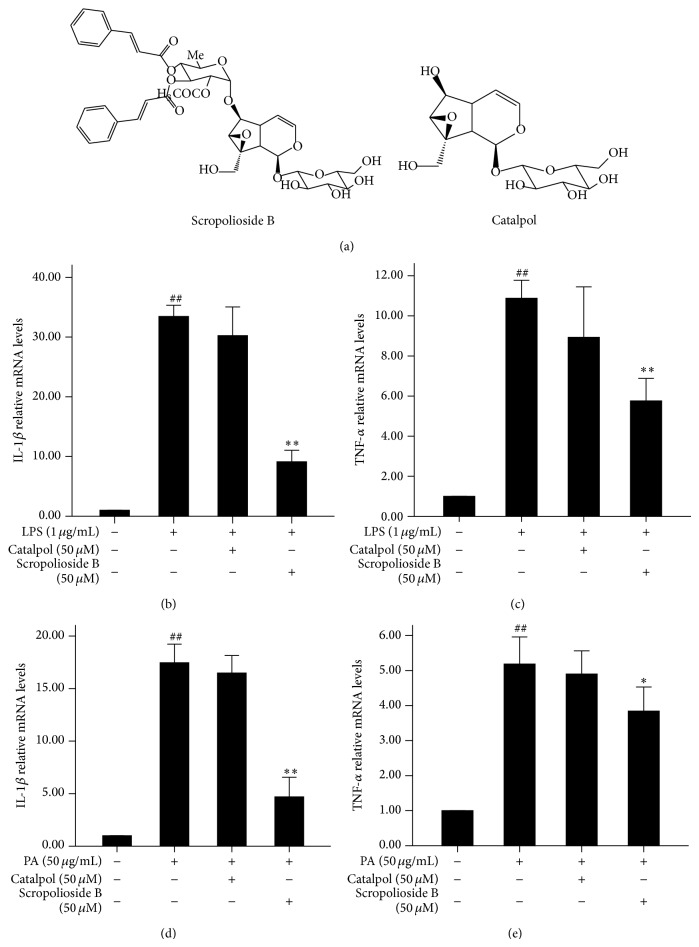
Different chemical structures of scropolioside B and catalpol. (a) The effects of scropolioside B and catalpol on LPS-induced expression of IL-1*β* and TNF-*α* in THP-1 cells. THP-1 cells were pretreated with 50 *μ*mol/L catalpol or scropolioside B for 1 h and then stimulated with LPS (1 *μ*g/mL) for another 24 h. ((b)-(c)) The effects of scropolioside B and catalpol on PA-induced expression of IL-1*β* and TNF-*α* in THP-1 cells. THP-1 cells were pretreated with 50 *μ*mol/L catalpol or scropolioside B for 1 h and then stimulated with PA (50 *μ*g/mL) for another 24 h. ((d)-(e)) The expression of IL-1*β* and TNF-*α* mRNA was measured by RT-PCR. The data represent the mean values of over three experiments ± SD. ^##^ *P* < 0.01 compared to vehicle control, ^**^ *P* < 0.01 compared to LPS or PA alone. ^#^ *P* < 0.05 compared to vehicle control, ^*^ *P* < 0.05 compared to LPS or PA alone.

Since scropolioside B contains structures of catalpol and two phenylpropanoids (Figure 1(a)), we compared and tested the anti-inflammatory capabilities of both scropolioside B and catalpol in THP-1 cells. The expression of IL-1*β* and TNF-*α* was significantly induced by lipopolysaccharide (LPS) or palmitic acid (PA), a free fatty acid with potential proinflammatory mediators, compared to control-treated THP-1 cells (Figure 1). This indicated that cellular exposure to LPS or PA induced the secretion of various cytokines that lead to the initiation and amplification of inflammation. To investigate the anti-inflammatory effect of scropolioside B, we preincubated THP-1 cells with the compound for 1 h and subsequently stimulated the cells with LPS or PA. We found that scropolioside B significantly blocked the increase in IL-1*β* and TNF-*α* levels induced by LPS or PA (Figure 1). However, at the concentration of 50 *μ*mol/L, catalpol did not effectively block expression of IL-1*β* and TNF-*α*, although these anti-inflammatory effects had been reported [24, 25]. These observations suggested that scropolioside B has stronger anti-inflammatory activity compared to catalpol.

### 3.2. Inhibition of Nuclear Factor *κ*B Activation by Scropolioside B

**Figure 2:**
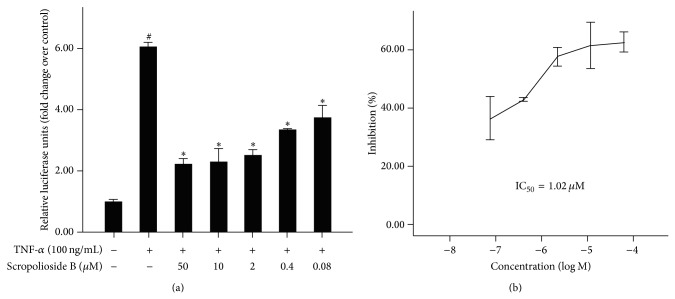
Scropolioside B inhibited TNF-*α*-induced NF-*κ*B activation. Cells were preincubated for 1 hour with different doses of scropolioside B and then stimulated with 1 *μ*g/mL TNF-*α* for 16 hours. The results shown are representative of 3 separate experiments. Data are expressed as means ± SD. ^#^ *P* < 0.05 versus the control, ^*^ *P* < 0.05 versus the TNF-*α* (a). IC_50_ values of 1.02 *μ*mol/L (b).

NF-*κ*B is an essential transcription factor involved in the production of several cytokines that mediate the inflammatory response. To investigate overall anti-inflammatory activity of scropolioside B, we used a luciferase reporter assay to determine nuclear factor kappa B (NF-*κ*B) activity. After HEK293 cells were transferred with either the NF-*κ*B or the control plasmid, cells were treated with or without scropolioside B for 1 h and then stimulated with 100 ng/mL of TNF-*α*. An increase in luciferase activity was observed after stimulation with TNF-*α*, suggesting that NF-*κ*B was activated by TNF-*α* (Figure 2(a)). Pretreatment with scropolioside B (0.08–50 *μ*mol/L) inhibited TNF-*α*-induced NF-*κ*B activation in a concentration-dependent manner. Furthermore, scropolioside B exhibited an IC_50_ value of 1.02 *μ*mol/L (Figure 2(b)). These results showed that scropolioside B-mediated inhibition of inflammatory cytokine induction was due to the suppression of NF-*κ*B.

### 3.3. Scropolioside B Reduced the IL-32 Expression

**Figure 3:**
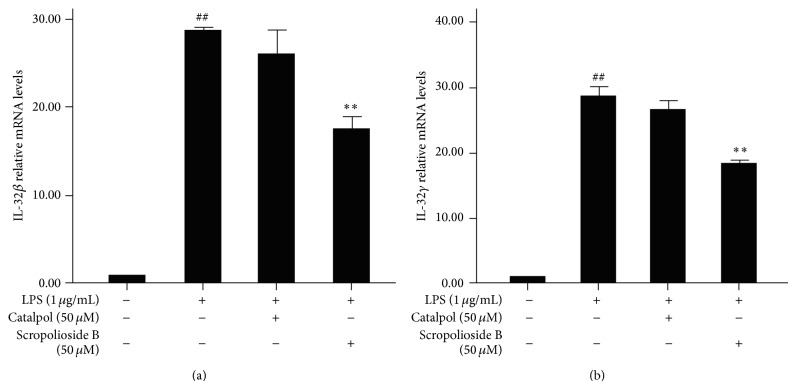
The effects of scropolioside B and catalpol on LPS-induced expression of IL-32*β* and IL-32*γ* in THP-1 cells. THP-1 cells were pretreated with 50 *μ*mol/L catalpol or scropolioside B for 1 h and then stimulated with LPS (1 *μ*g/mL) for another 24 h. The expression of IL-32*β* and IL-32*γ* mRNA was measured by RT-PCR. The data represent the mean values of over three experiments ± SD. ^##^ *P* < 0.01 compared to vehicle control, ^#^ *P* < 0.05 compared to vehicle control, ^**^ *P* < 0.01 compared to LPS alone, and ^*^ *P* < 0.05 compared to LPS alone.

IL-32 is a proinflammatory cytokine involved in several diseases, including infections, chronic inflammation, and cancer. TNF-*α* or LPS are known inducers of IL-32, IL-32-dependent effects of IL-1*β* on endothelial cell functions [26]. We next determined the inhibitory effects of scropolioside B on IL-32 expression. Pretreatment with scropolioside B significantly diminished the increase in mRNA expression levels of IL-32*β* and IL-32*γ* induced by LPS stimulation (Figures 3(a) and 3(b)), similar to IL-1*β* and TNF-*α* expression pattern, in LPS-induced THP-1 cells.

### 3.4. Scropolioside B Decreases Expression of NLRP3

**Figure 4:**
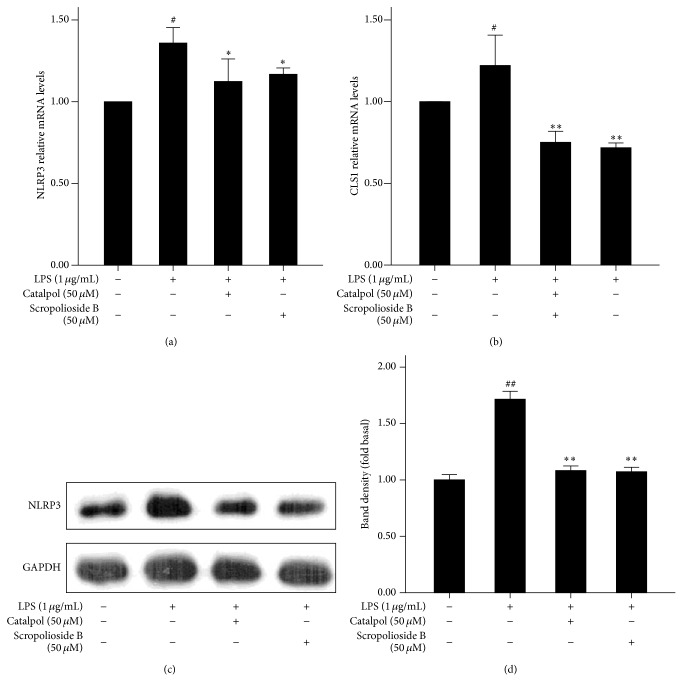
Scropolioside B and catalpol inhibited the expression of CLS1 and NLRP3 in LPS-induced THP-1 cells. ((a)-(b)) The effects of scropolioside B and catalpol on protein expression of NLRP3 in LPS-induced THP-1 cells. ((c)-(d)) THP-1 cells were pretreated with 50 *μ*mol/L catalpol or scropolioside B for 1 h and then stimulated with LPS (1 *μ*g/mL) for another 24 h. The protein expression of NLRP3 was measured by western blot. The expression of CLS1 and NLRP3 mRNA was measured by RT-PCR. The data represent the mean values of over three experiments ± SD. ^##^ *P* < 0.01 compared to vehicle control, ^#^ *P* < 0.05 compared to vehicle control, and ^**^ *P* < 0.01 compared to LPS alone.

Inflammasomes regulate maturation of IL-1*β* and IL-18 and pyroptosis. NLRP3 is a member of inflammasomes which constitute the compound with ASC and caspase-1 to catalyze the maturation of IL-1*β* [27]. To observe whether inflammatory factors induce NLRP3 expression, we stimulated THP-1 cells with LPS for 24 h. We observed that LPS upregulated NLRP3 mRNA and protein (Figures 4(a), 4(c), and 4(d)). Similarly, LPS also significantly enhanced mRNA expression of cardiolipin synthetase 1 (CLS1) (Figure 4(b)), a mitochondrial enzyme catalysing cardiolipin synthesis necessary for inflammasome NLRP3 activity [28]. As shown in Figures 4(a)–4(d), pretreatment with scropolioside B inhibited the expressions of NLRP3 mRNA and protein, as well as CLS1 mRNA. We also found that catalpol was as equally effective as scropolioside B (Figures 4(a)–4(d)), suggesting that this inhibitory effect may be from the same catalpol structure (Figure 6).

### 3.5. Scropolioside B Decreases Expression of Pro-IL-1*β* and IL-1*β*

**Figure 5:**
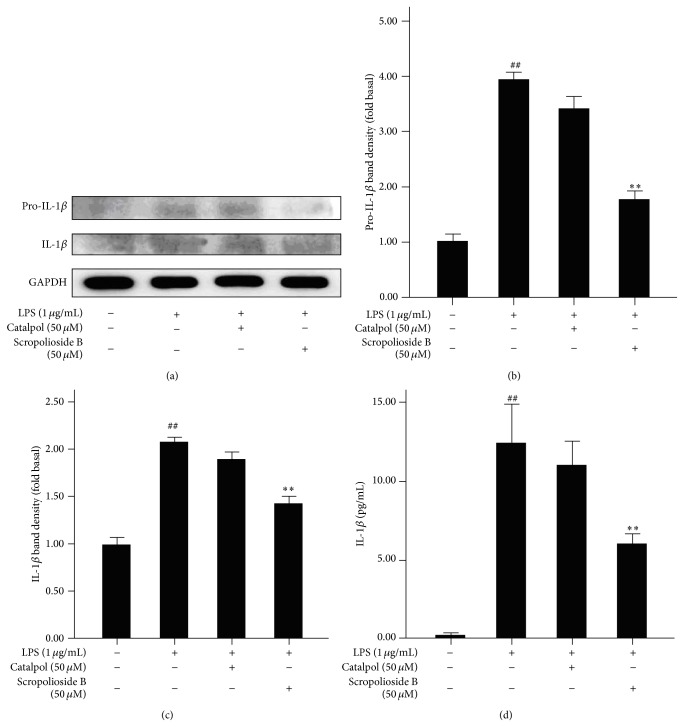
Scropolioside B and catalpol inhibited the expression of pro-IL-1*β* and IL-1*β* in LPS-induced THP-1 cells. ((a)–(d)) THP-1 cells were pretreated with 50 *μ*mol/L catalpol or scropolioside B for 1 h and then stimulated with LPS (1 *μ*g/mL) for another 24 h. The protein expression of pro-IL-1*β* and IL-1*β* was measured by western blot. The levels of cytokines in the medium were measured using an ELISA kit. The data represent the mean values of over three experiments ± SD. ^##^ *P* < 0.01 compared to vehicle control, ^**^ *P* < 0.01 compared to LPS alone.

**Figure 6:**
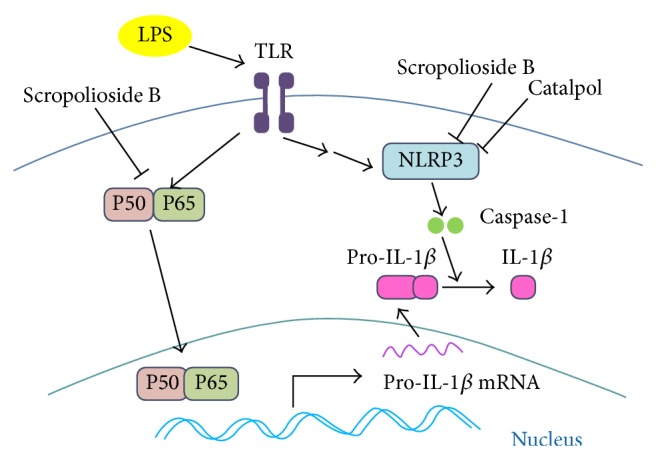
A schematic diagram of the regulation of NLRP3 inflammasome by scropolioside B and catalpol.

To comprehensively evaluate the inhibitory effect of scropolioside B and catalpol, we performed further studies about scropolioside B impact on protein expression of pro-IL-1*β* and IL-1*β*. As shown in Figures 5(a)–5(d), pretreatment with scropolioside B inhibited the expressions of pro-IL-1*β* and IL-1*β* protein. The inhibition of pro-IL-1*β* is stronger than IL-1*β*. Catalpol does not inhibit the protein expression of pro-IL-1*β* and IL-1*β*.

## 4. Discussion

Scropolioside B which is from* S. dentata* Royle ex Benth. has antipyretic detoxicating effect. It is used in Tibetan medicine, such as smallpox, measles, other infectious fevers, and inflammatory diseases. In this study, we demonstrated that scropolioside B, an iridoid glycoside containing a catalpol and two phenylpropanoids, was more effective than catalpol in inhibiting the expressions of IL-1*β* and TNF-*α* in THP-1 cells activated by LPS or palmitic acid (Figure 1). Our results suggested that scropolioside B has higher anti-inflammatory activity than catalpol, although some studies have reported that catalpol does demonstrate anti-inflammatory effects at high dose by inhibiting COX-2 activity, TNF-*α* formation, monocyte chemotactic protein-1 (MCP-1), and nitric oxide production [24, 25]. As shown in Figure 2, the anti-inflammatory effects of scropolioside B were mediated by blocking NF-*κ*B activity (Figure 2). IL-1*β* is involved in the onset of acute local or systemic inflammation and contributes to a variety of chronic noninfectious diseases, including ischemic injury, atherosclerosis, type 2 diabetes, and osteoarthritis. Endogenous metabolites, such as oxidized fatty acids, high glucose, uric acid crystals, activated complement, necrotic cells, and cytokines, can stimulate the synthesis of the inactive IL-1*β* precursor, which awaits processing by the inflammasome complex to be activated [29].

Inflammasomes regulate maturity of IL-1*β*, IL-18, and pyroptosis and recognize microbial products or endogenous molecules released from damaged cells [27]. Inflammasomes have several member proteins, including NLRP1, NLRP3, NLRC4, AIM2, and NRP6 [6]. Our results showed that scropolioside B can inhibit mRNA and protein expression of inflammasome NLRP3 and prevents the secretion of IL-1*β* (Figure 4). Interestingly, catalpol has a similar inhibitory effect on NLRP3 expression compared to scropolioside B, suggesting that scropolioside B and catalpol inhibit IL-1*β* and NLRP3 expression by different mechanisms. Scropolioside B and catalpol also block the expression of CLS1, which is an enzyme in the final step of mitochondrial cardiolipin synthesis by catalysing the transfer of a phosphatidyl residue from diacylglycerol to phosphatidylglycerol. Iyer et al. [28] reported that mitochondrial cardiolipin and reactive oxygen species are needed for inflammasome NLRP3 activity. Cardiolipin can bind directly to NLRP3 and silencing of cardiolipin synthesis specifically inhibits inflammasome NLRP3 activation [28]. Based on these observations, we believe that scropolioside B not only blocks NF-*κ*B pathway but also inhibits NLRP3, CLS1, and IL-1*β* expressions. However, catalpol only prevents the expression of NLRP3 and CLS1 (Figure 5).

Our results also showed that scropolioside B, but not catalpol, blocked IL-32*β*/*γ* expression (Figure 3). Several studies have shown that IL-32, an important proinflammatory cytokine in rheumatoid arthritis, enhanced IL-6 and IL-8 production in fibroblast-like synoviocytes [30–32]. Some studies also have shown that IL-32 is closely associated with liver fibrosis of chronic viral hepatitis [33, 34]. Furthermore, compared with primary blood monocytes, IL-1*β*, TNF-*α*, or LPS can stimulate high levels of IL-32 expression through the I*κ*B kinase-*β*/NF-*κ*B and ERK pathways in human umbilical vein endothelial cells, aortic macrovascular endothelial cells, and cardiac and pulmonary microvascular endothelial cells [26]. Conversely, IL-32 also stimulates IL-1*α*, IL-1*β*, IL-6, TNF-*α*, and chemokines via NF-*κ*B, p38 MAPK, and AP-1 activation [26, 35]. IL-32 promotes angiogenesis propagating vascular inflammation and exacerbates sepsis in a mouse model [36, 37]. Recent studies have shown that atherosclerosis maybe associated with IL-32 production [38].

In conclusion, scropolioside B significantly diminished expression and secretion of IL-1*β*, IL-32, and TNF-*α*. We show that this is mediated by modulating NF-*κ*B, NLRP3, and CLS1 levels. Additional studies are needed to further elucidate other targets by which scropolioside and catalpol regulate inflammation. The results of this study strengthen previous understanding of the anti-inflammatory effects of iridoids and highlight scropolioside B as a potential drug for the treatment of rheumatoid arthritis and atherosclerotic disease.

## Figures and Tables

**Table 1:** Primer sequences of the genes tested in this study.

Gene	Direction	Primer sequences
IL-1*β*	Forward	5-AAACAGATGAAGTGCTCCTTCCAGG
	Reverse	5-TGGAGAACACCACTTGTTGCTCCA

TNF-*α*	Forward	5-CAGAGGGAAGAGTTCCCCAG
	Reverse	5′-CCTTGGTCTGGTAGGAGACG

IL-32*β*	Forward	5-GAGTTTCTGCTGCTCTCTGTCA
	Reverse	5-ATTTTGAGGATTGGGGTTCAG

IL-32*γ*	Forward	5-GAGTTTCTGCTGCTCTCTGTCA
	Reverse	5-ATTTTGAGGATTGGGGTTCAG

NLRP3	Forward	5-CTACACACGACTGCGTCTCATCAA
	Reverse	5-GGGTCAAACAGCAACTCCATCTTA

CLS1	Forward	5-GAGTATGCCACAGTATGAAAACCCA
	Reverse	5-CGAGCAATAAATCCATCCAACAA

GAPDH	Forward	5-AGAAGGCTGGGGCTCATTTG
	Reverse	5-AGGGGCCATCCACAGTCTTC
